# Application of metagenomic next-generation sequencing in the diagnosis of urinary tract infection in patients undergoing cutaneous ureterostomy

**DOI:** 10.3389/fcimb.2023.991011

**Published:** 2023-01-27

**Authors:** Rong Huang, Qian Yuan, Jianpeng Gao, Yang Liu, Xiaomeng Jin, Liping Tang, Ying Cao

**Affiliations:** ^1^ Nursing Department, The First Affiliated Hospital of Nanchang University, Nanchang, China; ^2^ Medical department, Genskey Medical Technology Co., Ltd, Beijing, China; ^3^ Clinical Laboratory, The First Affiliated Hospital of Nanchang University, Nanchang, China; ^4^ Thoracic Surgical ICU, Yantai Yuhuangding Hospital, Yantai, China

**Keywords:** UTI, mNGS, cutaneous ureterostomy, biomarker, ARGs

## Abstract

**Objective:**

Urinary tract infection (UTI) is an inflammatory response of the urothelium to bacterial invasion and is a common complication in patients with cutaneous ureterostomy (CU). For such patients, accurate and efficient identification of pathogens remains a challenge. The aim of this study included exploring utility of metagenomic next-generation sequencing (mNGS) in assisting microbiological diagnosis of UTI among patients undergoing CU, identifying promising cytokine or microorganism biomarkers, revealing microbiome diversity change and compare virulence factors (VFs) and antibiotic resistance genes (ARGs) after infection.

**Methods:**

We performed a case-control study of 50 consecutive CU patients from December 2020 to January 2021. According to the clinical diagnostic criteria, samples were divided into infected group and uninfected group and difference of urine culture, cytokines, microorganism, ARGs and VFs were compared between the two groups.

**Results:**

Inflammatory responses were more serious in infected group, as evidenced by a significant increase in *IFN-α* (p=0.031), *IL-1β* (0.023) and *IL-6* (p=0.018). Clinical culture shows that there is higher positive rate in infected group for most clinical pathogens like *Escherichia coli, Klebsiella pneumoniae, Staphylococcus aureus, Candida auris etc.* and the top three pathogens with positive frequencies were *E. coli*, *K. pneumoniae*, and *Enterococcus faecalis.* Benchmarking clinical culture, the total sensitivity is 91.4% and specificity is 76.3% for mNGS. As for mNGS, there was no significant difference in microbiome α- diversity between infected and uninfected group. Three species biomarkers including *Citrobacter freundii, Klebsiella oxytoca*, and *Enterobacter cloacae* are enriched in infected group based on Lefse. *E. cloacae* were significantly correlated with IL-6 and IL-10. *K. oxytoca* were significantly correlated with IL-1β. Besides, the unweighted gene number and weighted gene abundance of VFs or ARGs are significantly higher in infected group. Notablely, ARGs belonging to fluoroquinolones, betalatmas, fosfomycin, phenicol, phenolic compound abundance is significantly higher in infected group which may have bad effect on clinical treatment for patients.

**Conclusion:**

mNGS, along with urine culture, will provide comprehensive and efficient reference for the diagnosis of UTI in patients with CU and allow us to monitor microbial changes in urine of these patients. Moreover, cytokines (*IL-6, IL-1β*, and *IFN-a*) or microorganisms like *C. freundii, K. oxytoca* or *E. cloacae* are promising biomarkers for building effective UTI diagnostic model of patients with CU and seriously the VFs and ARGs abundance increase in infected group may play bad effect on clinical treatment.

## Introduction

With estimated 573,000 new cases and 213,000 deaths in 2020, bladder cancer is the tenth most dominating cancer worldwide (Sung et al., 2021). Several factors, such as tobacco smoking and exposure to certain chemicals, have been regarded as main risk factors contributing to bladder cancer (Cumberbatch et al., 2018). Radical cystectomy (RC) with urinary diversion (UD) is the gold standard for the treatment of muscle invasive bladder cancer (MIBC) and high-risk non-muscle invasive bladder cancer (NMIBC) (Farber et al., 2018). Cutaneous ureterostomy (CU), a reliable choice for the older and frail population, has its advantage like relatively simple procedure and faster postoperative recovery time (Witjes et al., 2021).

Nowadays, Urinary tract infection (UTI) is the second most common infectious disease, caused by different type of microbial agents, which can stimulate innate and adaptive responses passing through the physical barriers. Among these responses, toll-like receptors (TLRs), which activate a range of different immune system components, including chemokines, interferons, interleukins, antimicrobial peptides, and proinflammatory cytokines, are critical component of the innate immune response to all types of infection (Behzadi and Behzadi, 2016). UTI is a common complication for patients undergoing RC with an incidence ranging from 19% to 43% (Mano et al., 2014; Ghoreifi et al., 2020; Gayarre Abril et al., 2021). In addition, patients with CU also need to be inserted with ureteral stents to drain urine, and research has shown that a bacterial biofilm can be formed on the surface of these stents in only a short space of time. Consequently, it is difficult for antibiotics to enter, thus leading to catheter-related UTI, drug resistance, and prolonged UTI (Bader et al., 2013). Recurrent UTI and increasing antimicrobial resistance lead to an increase in readmission rate and economic burden of patients, thus exerting serious effects on a patient’s life quality (Flores-Mireles et al., 2015; Liu et al., 2016). Therefore, patients with CU need efficient and precise diagnosis in order to be treated effectively as early as possible and to avoid overtreatment.

However, diagnosis of UTI is generally based on traditional urinary culture tests and clinical manifestations; these strategies are associated with certain limitations. First, traditional urinary culture tests are suitable for fast-growing bacteria, whereas some organisms not suitable for standard culture conditions such as fastidious bacteria and fungi are involved in UTI (Siddiqui et al., 2011; Wolfe et al., 2012; Moustafa et al., 2018). Second, traditional urine culture and drug sensitivity tests take a long time to complete and cannot guide the selection of clinical antibiotics in a rapid manner. Third, bladder is not sterile, and studies of the urine of healthy subjects has provided evidence of bladder-associated microbial populations (Smelov et al., 2016). Moreover, patients undergoing CU whose bladder is removed, do not have typical symptoms of UTI such as frequent urination, urgency, and dysuria. In addition, indwelling ureteral stents can lead to a high incidence of bacteriuria. It has been reported that the incidence of chronic indwelling stent bacterial colonization and bacteriuria is 100% (Kaufman et al., 2009), which all complicate the diagnosis of UTI in these patients.

Metagenomic next-generation sequencing (mNGS) is an unbiased method to sequence all nucleic acids (DNA) of a specimen in parallel (Simner et al., 2018). It can be used to accurately detect a wide range of pathogens. This emerging approach is favored by clinical laboratories for pathogenic detection of infectious diseases and has been used to determine the etiological diagnosis of some infectious diseases (Simner et al., 2018). Formerly, a positive urine culture test was considered to be indicative of UTI or asymptomatic bacteriuria (Whiteside et al., 2015). As urinary tract of healthy subjects has its own unique flora structure, mNGS can provide more comprehensive data relating to the microenvironment change of a patient’s urinary tract than traditional culture. This may help doctors locate organisms that cause disease more accurately and move towards a “precision medicine” model (Dixon et al., 2020). In addition, mNGS facilitates mining VFs and ARGs so as to monitor the prevalence of highly virulent and drug-resistant microorganisms.

In the study, focusing on patients undergoing CU, we aimed to explore the performance of mNGS in helping diagnosis UTI, the significantly different biomarkers such as cytokines, microorganism structure and pathogenic microorganisms which would lay a foundation for future UTI diagnostic model construction.

## Materials and methods

### Patients and samples

We performed a case-control study of 50 patients with CU from December 2020 to January 2021. Skin swabs around the stoma, ureteral stents, urine and blood samples were collected according to the standard procedure (National Health Commission of the people's Republic of China, 2018). Urine, ureteral stents and skin swabs were collected for routine culture test and urine samples were frozen in the freezer at -80°C and sent to Genskey’s laboratory for mNGS test. Additionally, cytokines detection and urinalysis were performed. All samples were submitted for inspection immediately after collection. According to the inclusion-exclusion criteria and clinical infection diagnostic criteria (**Table 1**) (Horan et al., 2008; National Health and Family Planning Commission of the people's Republic of China, 2016; Flaig et al., 2020), the included patients were classified into an infected group and an uninfected group.

**Table 1:** Several criteria in our study.

Inclusion criteria	
1)	Patients who were diagnosed as bladder cancer and underwent CU according to the bladder cancer diagnosis and treatment guidelines of the National Comprehensive Cancer Network (NCCN) in 2020
2)	Patients who gave informed consent to participate in this study
Exclusion criteria	
1)	Patients who were complicated with infection besides urinary system
2)	Patients who were on long-term steroids and immunosuppressants
3)	Patients who received antibiotics treatment within 14 days prior to inclusion
Elimination criteria	
1)	The urine samples were sequenced by mNGS and suspected of contamination or had too few sequences
Clinical infection diagnostic criteria	
1)	A positive urine culture (≥10^5^cfu/mL) with documented symptoms (fever, flank pain, changes in urine color, character or smell, etc.)
2)	A positive urine culture (≥10^5^cfu/mL) that need to receive antibiotic treatment by practitioner discretion
3)	A negative/unavailable urine culture with documented symptoms consistent with a clinical diagnosis of UTI (including urinalysis suggestive of UTI)

### Urinalysis

It was detected by AX4030 automatic urine dry chemical analyzer (Arkray Company of Japan). Urine bacterial count and white blood cell count were determined by UF1000i urine tangible component analyzer (Sysmex Company, Japan), and the operation was carried out strictly in accordance with the instrument instructions.

### Urine culture and susceptibility testing

1μL clean midstream urine was inoculated on Columbia blood plate and McConkey medium plate (Oxoid company, UK). The isolated bacteria were counted after cultured at 37°C for 48 hours. Bacterial identification and drug sensitivity test were carried out by VITEK 2 Compact automatic identification drug sensitivity instrument (Bio Mérieux S.A.). According to the Clinical and Laboratory Standards Institute (CLSI) guidelines (Humphries et al., 2018), MICs were admeasured using frozen Trek Sensititre custom plates (Thermo Scientifific).

### Cytokines detection

Detection of 12 cytokines by flow immunofluorescence photoluminescence with multiple microspheres. The kit was purchased from Qingdao Raiscare Biotechnology Co., Ltd., and the detection instrument was BD FACS Calibur flow cytometry.

### DNA extraction, library preparation and mNGS

Urine samples (5mL) and the negative batch controls samples were collected using Sterile screw freezing tubes. 800uL was absorbed from the liquefied sample, transferred to centrifuge tube, and centrifuged at 13600g for 5min. the supernatant was discarded, and the precipitation was used for extraction. DNA was extracted with a Genskey Micro DNA Kit (1901, Genskey, Tianjin) and measured by Qubit dsDNA HS Assay Kits.

The DNA libraries were constructed by an NGS library construction kit(2012B, Genskey, Tianjin). The DNA libraries quality was assessed using an Agilent 2100 Bioanalyzer (Agilent Technologies, SantaClara, USA). The constructed DNA library concentration was determined by qPCR and, must be at least 1 Nmol/L.

Finally, all sample DNA libraries were mixed and sequenced with a single-stranded circular DNA was added by 2-3 quantitative sets to obtain DNA nanospheres. The DNA nanospheres were loaded on the sequencing chip and sequenced using the MGISEQ-2000 sequencing platform MGI, Shenzhen, China).

### Bioinformatic analysis of mNGS

#### Data quality control.

For quality control like adapter contamination and low-quality, raw reads were filtered by fastp (v0.19.5) (Humphries et al., 2018). Reads that were mapped to human reference assembly GRCh38 were removed with bowtie2 v2.3.4.3 (Langmead and Salzberg, 2012).

#### Taxonomic assignment and comparation with culture

After that reads were aligned to the microorganism database consisted of about 12000 genomes (including bacteria, viruses, fungi, protozoa, homo sapiens and other multicellular eukaryotic pathogens) (Jing et al., 2021) with kraken2 (v2 2.1.2) (Lu et al., 2022) as previously described. Then, the abundance of the annotated taxa was corrected based on bracken (-r 50 -t 10) (Lu et al., 2017) and was further standardized to 20,000,000 (20M) sequencing data. In order to determine the positive detection rate of mNGS, we calculated the ratio of the abundance of each pathogen in the samples versus the controls in the same batch. If foldchange >10 or pathogens were not detected in the controls, positive mNGS was defined (Miller et al., 2019). Next, the sensitivity, specificity and accuracy of each pathogen were calculated in urine samples positive for the clinical culture. Sensitivity was defined as TP/(TP+FN). Specificity was defined as TN/(TN+FP). Accuracy was defined as (TP+TN)/(TP+FP+TN+FN).

#### Microbiome diversity comparation.

To assess the organism richness rarefaction analysis was performed with in-house R script on species level. We also calculated the within-sample (α) diversity using Shannon index and Simpson index on genus level to estimate the richness of samples using QIIME v1.9.1 (Hall and Beiko, 2018). Principal Coordinate Analysis (PCoA) ware performed using Bray-Curtis distance.Permutational analysis of variance (PERMANOVA) was applied to test group effection like “Gender”, “Chronic Underlying Diseases”, “Batch effect”, “Infected or Uninfected “ based on distance matrix using vegan package(distance = ‘bray’, permutations = 999) (Wang et al., 2020). LefSe (Linear discriminant analysis Effect Size) (Segata et al., 2011) was used to test the difference in taxa abundance and function analyze. To evaluate their associations between microorganisms and inflammatory factors, we performed mantel test analyze to find the possible biomarker (Yang et al., 2017).

#### VFs comparation.

We analyzed bacterial virulence factors using Virulence factor database (VFDB) (Chen et al., 2005) by BLASTn v2.9.0+ (Chen et al., 2015) based method with 95% identity and 95% query coverage cutoffs, relatively strict than previous research (Jin et al., 2021). VFs abundance were calculated as follows (Zhao et al., 2020):


$$\text{Abundance }(coverage,/M\;read)\;=\;\frac{(N_{mapped\;reads}*L_{reads})/L_{VFs}}{\text{S}}$$

Where N_mapped reads_ is the number of reads mapped to VF and L_reads_ is the sequence length of BGI reads (SE50). L_VFs_ is the length of VFs and S is the size of the data (M read)

Next, we conducted a comparison of the total gene number and abundance of the VF among the infected group, uninfected group and controls as presented in boxplot figure and heatmap. A differential VF analysis was performed among three groups *via* Lefse Then, we combined the differential VFs with the classification of VF and contribution of taxa accordint to VFDB database and showed the relationship by Sankey diagram. Box figure and heatmap were visualized through R pheatmap package. Sankey diagram were visualized through https://sankeymatic.com/.

#### ARGs comparation.

We performed read-based method to detect ARGs by RGI bwt model (Alcock et al., 2020) and calculated the ARG abundance in the same way as VFs. Total ARG gene number and ARG abundance were compared among the infected, uninfected and NC group with boxplot. Procrustes analysis (Wang et al., 2020) was performed to evaluate the correlation between ARG profiles and species community structure on the base of Bray-Curtis matrices. Drug class is from the output file of RGI with further manual integration.

### Statistical analysis

Power Analysis was performed by pwr.t2n.test function in pwr (v1.2-2) package of R. Odds ratio analyze by R package epitools oddsratio function. The Wilcoxon test (Mann Whitney U-test) was used to analyze differences across subgroups which was performed by R ggpubr package. Data analysis was performed by GraphPad Prism 8 software and R. P< 0.05 was statistically significant in our study.

### Code availability

The core software used is described in Materials and Methods. The scripts in this study are available on github: https://github.com/GoGoGao/UTI_mNGS_research.

## Result

### Subject characteristics and study design

Our study protocols were in compliance with the published research (Chen et al., 2020). In the group with underlying diseases, the incidence of infection was 77.8% (14/18) while in the group without underlying disease, the incidence of infection was only 65.5% (21/32) (**Supplementary Figure 1A**). The odds ratio of underlying disease to no underlying disease was 1.82 (95% CI = [0.42,9.41], P=0.39). Though we observed a ratio increase of the Infected and Uninfected, it was not statistically significant. As bladder cancer is more prevalent in males,^1^ our study featured more male patients (42/50, 84%) than female patients (8/50, 16%). When all 42 male cases were considered, we found that 71.4% (30/42) had infection; this was higher than the rate of infection in females (5/8; 62.5%) (**Supplementary Figure 1B**). The odds ratio of male to female is 0.67 (95% CI = [0.11,5.01], P=0.63). There was no significant difference between the two groups with regards to age (P=0.24, Wilcoxon test).

When compared to the uninfected group by Wilcoxon, the serum levels of *IFN-α* (p=0.031), *IL-1β* (0.023), *IL-6* (p=0.018), *LE* (p=1.9e-05), along with *U_BACT* (p=0.00016) and *WBC count* (p=0.00016), were all significantly higher in infected group (**Supplementary Figure 2**). Although no statistical difference was evident, the levels of *IFN-γ*, *IL-17*, along with *red blood cell (RBC)*, in the infected group also showed a tendency to be higher in infected group (**Table 2**). Therefore, inflammatory responses were more serious in infected group, probably due to the presence of pro-inflammatory bacteria. Boxplot analysis of the 3 key significantly different cytokines between infected and uninfected revealed that their concentrations increased with disease exacerbation.

**Table 2:** Characteristics of subjects.

Patients Characteristics	Uninfected (n=15)	Infected (n=35)	P	Normal Range	Characteristics type
Gender			–		**basic**
Male	12 (80%)	30 (86.0%)			
Female	3 (20%)	5 (14.0%)			
Age (years)	64 (47-80)	67 (57-82)	0.240		
IFN-a, pg/ml	1.43 (0.31-2.5)	1.83 (0.78-3.18)	**0.031**	0-8.5	**Cytokines detection**
IL-1β, pg/ml	1.77 (0-23.25)	8.97 (0-73.67)	**0.023**	0-12.4	
IL-6, pg/ml	4.00 (0.1-9.1)	8.30 (0.25-30.95)	0.018*	0-5.4	
IFN-γ, pg/ml	6.10 (2.18-11.91)	6.50 (0.44-21.08)	0.920	0-23.1	
IL-10, pg/ml	0.83 (0.56-1.27)	0.91 (0.3-4.23)	0.700	0-12.9	
IL-12P70, pg/ml	0.21 (0-2.1)	0.06 (0-0.62)	0.550	0-3.4	
IL-17, pg/ml	1.57 (0.77-5.53)	2.08 (0.92-14.62)	0.120	0-21.4	
IL-2, pg/ml	0.61 (0-0.62)	0.77 (0-2.99)	0.480	0-7.5	
IL-4, pg/ml	0.64 (0.45-1.54)	0.63 (0-1.34)	0.140	0-8.56	
IL-5, pg/ml	1.98 (0.21-6.91)	2.14 (0-4.96)	0.380	0-3.1	
IL-8, pg/ml	8.72 (2.55-35.41)	9.31 (0-80.96)	0.320	0-20.6	
TNF-a, pg/ml	1.25 (0.04-4.35)	1.48 (0-7.36)	0.910	0-16.5	
Nitrite (NIT)	0 (0)	0.21 (0-1)	0.100	Negative (-)	**Urinalysis**
Leukocyte esterase (LE)	0.86 (0.5-2)	2.54 (1-4)	**0.000**	Negative (-)	
Bacteria count (U BACT), number/μl	179.82(0-881.8)	2613.64 (26.6-22218.4)	**0.000**	0-32.8	
Red blood cell count (RBC), number/μl	631.79(4.3-4729.7)	898.67 (0.1-9840.5)	0.750	0.2-13.8	
White blood cell count (WBC), number/μl	191.99(14.3-385.5)	2823.35 (7.5-36212.4)	0.000	0-7.1	

Data are presented as n (%) or means (range).

Bold and underlined number means P<0.05, Wilcoxon test.

**Figure 1:**
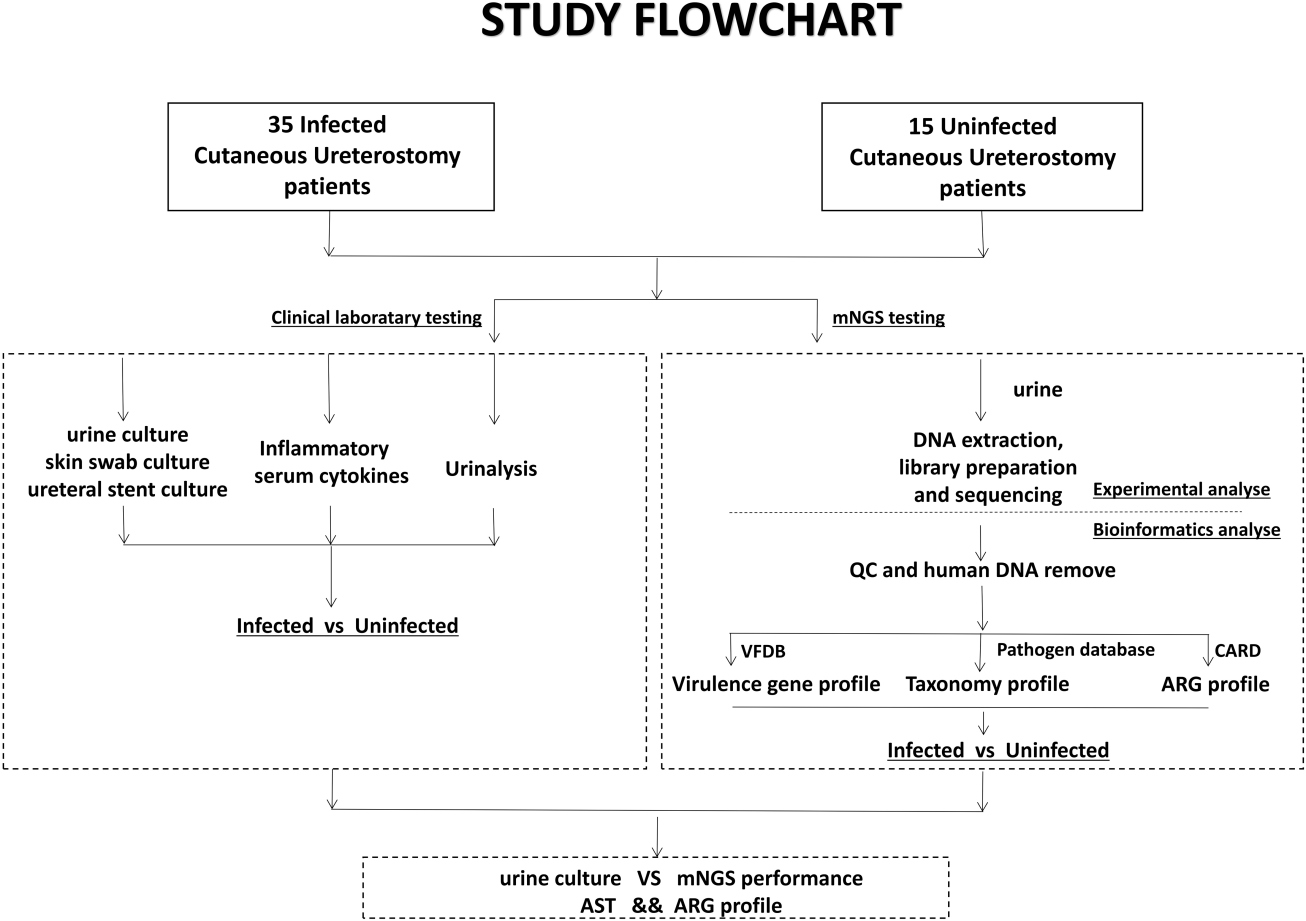
Schematic workflow of this study. Samples from 50 Cutaneous Ureterostomy patients were collected and sent for both conventional clinical laboratory testing and mNGS testing. We did Infected-vs-Uninfected difference analyse of urine culture, Inflammatory serum cytokines, virulence gene, taxonomy and ARG profile. Besides, comparation between urine culture and mNGS and association between ARG profile and Antibiotic Susceptible Test are performed.

Urine samples obtained from the patients were sent for both clinical laboratory tests and mNGS testing. The mNGS test pipeline included cfDNA (cell-free DNA) extraction from urine, library preparation, sequencing, and comprehensive bioinformatics analysis relating to virulence genes, taxonomy and ARG profile (**Figure 1**).

### The distribution of pathogens in patients undergoing CU

**Figure 2:**
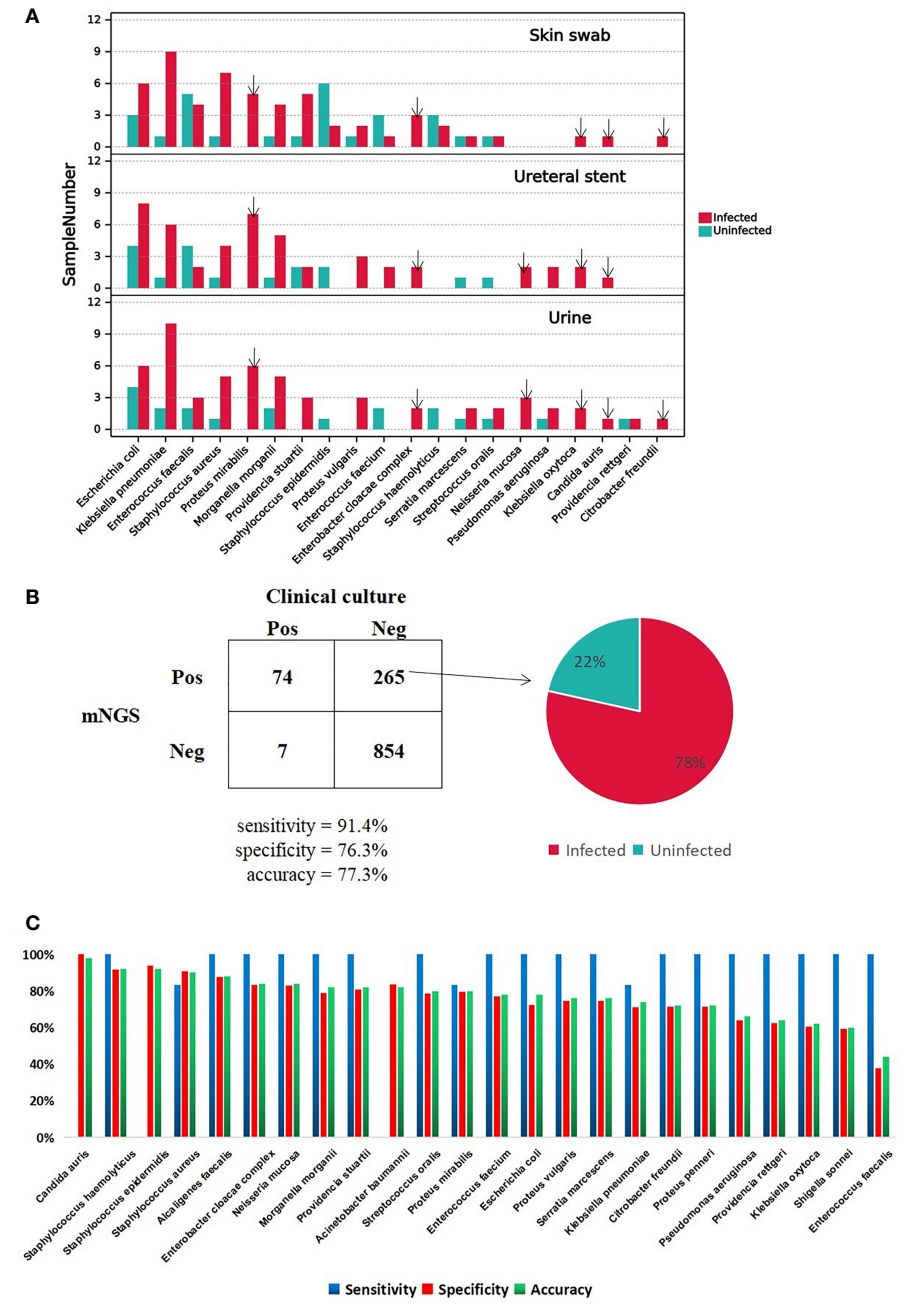
**A** Major positive microorganism of Clinical culture in Skin swab, Ureteral stent and Urine. The arrow marks the species detected only in Infected group. **B** The comparison of performance between Clinical culture and mNGS. The pie chart shows the proportion of false-positive microorganism identified by mNGS in Infected group versus uninfected group. **C**. The mNGS performance of each pathogen comparing with clinical culture.

To investigate the important pathogens leading to UTI in patients with CU, we performed traditional cultures on samples of urine, skin swabs, and ureteral stents, from 50 patients. The detailed culture results from each sample are shown in the table below (**Supplementary Table S1**). We also calculated the frequency of the positive microorganisms in all types of samples and ploted a column chart of 20 species which are culture positive at least in 2 samples in order to show differences between the two groups directly (**Figure 2A**). Analysis showed that the top three pathogens with positive frequencies in the three types of samples were *E. coli*, *K. pneumoniae*, and *E. faecalis* (**Supplementary Table S2**). Of these, the positive number of 17 species in the infected group was higher than that in the uninfected group, including *E. coli*, *K. pneumoniae*, *S. aureus*, *Proteus mirabilis*, and *Morganella morganii*. Six pathogens were positive only in the infected group: *P. mirabilis*, *E. cloacae complex*, *Neisseria mucosa*, *K. oxytoca*, *C. auri*, and *C. freundii* (**Figure 2A**). The presence of pathogens in uninfected group, including *E. coli*, *K. pneumoniae*, and *E. faecalis*, indicated that bacterial colonization also exists in normal urine. Therefore, we speculate that when the body’s immune system is weakened, these pathogens can reproduce and cause infection. Interestingly, except for *Providencia rettger*, the other 19 microorganisms are all positive whether in urine, skin swabs or ureteral stents, which indicates that the pathogenic organisms in urine may easily originate from their surrounding environment for patients with CU.

### mNGS performance comparing with clinical culture

DNA was extracted from urine samples and sequenced on the MGISEQ-2000 platform; 142.28 Gb of 50-bp single-end reads were generated in total, with an average of 45.17 ± 19.00 (standard deviation) million reads per sample. The data set for all samples was greater than 20M, which is a common data volume standard in mNGS research (**Supplementary Table S3**) (Liu et al., 2021). Based on foldchange >10 criteria for mNGS, mNGS exhibited high positive detection rate for pathogens in urine samples. Overall, the mNGS assay showed 91.4% sensitivity and 76.3% specificity compared to clinical culture (**Supplementary Table S4**, **Figure 2B**). The reason for the low specificity may be the negative results of clinical culture. In total 265 cases were classified as mNGS false-positive, and 78% of cases happen in the infected group (**Supplementary Table S5**; **Figure 2B**). There were four species with accuracy above 90%, *C. auris, Staphylococcus epidermidis, Staphylococcus haemolyticu* and *S. aureus*, and *C. auris* with the highest accuracy of 98%. The sensitivity, specificity and accuracy for each clinically culture positive pathogens are specifically shown in **Figure 2C**.

In a word, mNGS showed possibility to help clinician diagnose UTI but it is still a challenge to distinguish between colonizing bacteria and infectious bacteria whether for mNGS or culture.

### Microbiome diversity change

**Figure 3:**
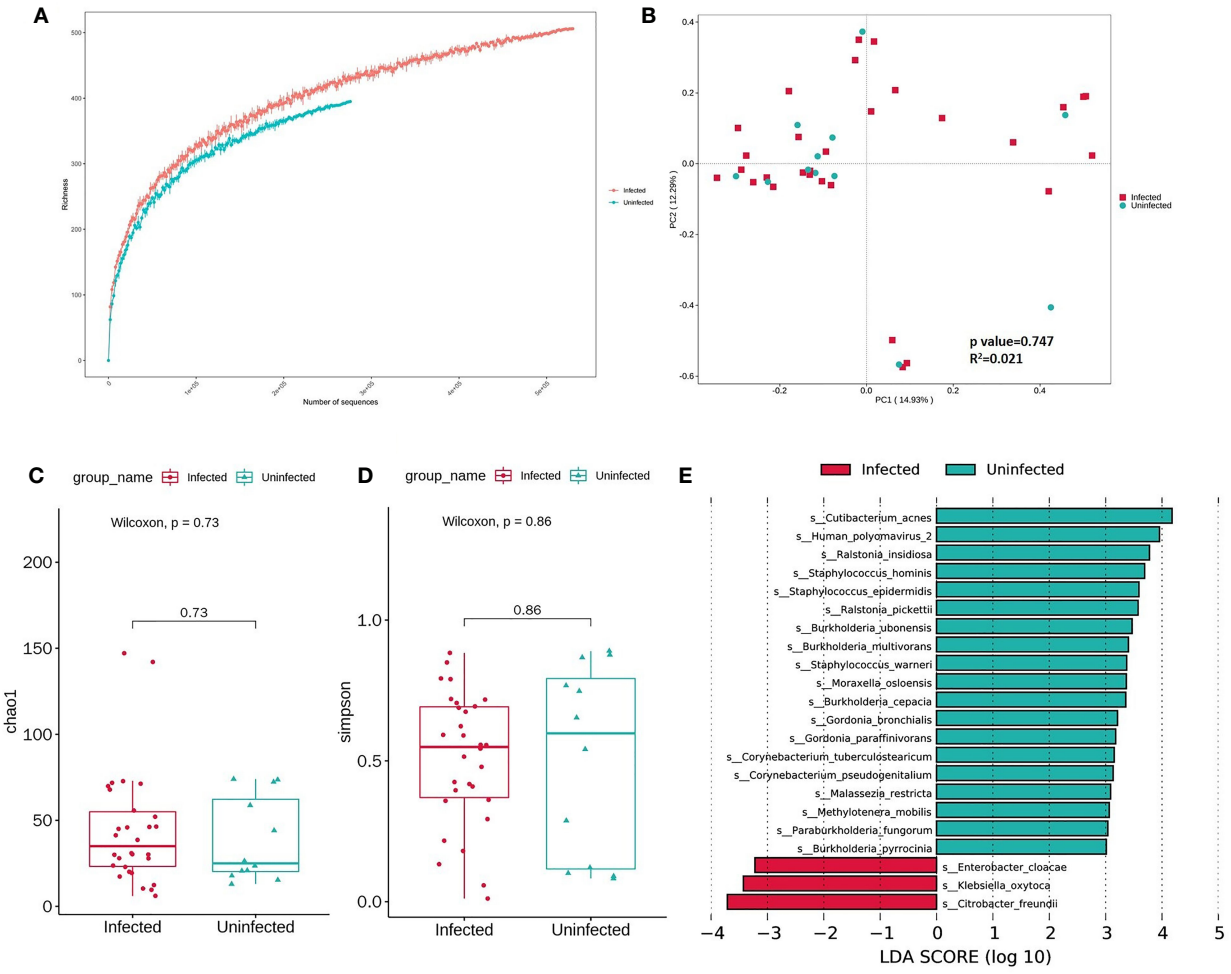
**(A)** Rarefaction curves for species number in Infected(n=35) and Uninfected (n = 15) after 100 random sampling. The curve in each group is near smooth when the sequencing data are great enough with few new species undetected. **(B)** Weighted Principal Coordinate Analysis (PCoA) using Bray-Curtis distance based on the relative abundance in 50 samples. Significant differences across groups are established at the first principal component (PC1) values and PC2, and shown in the box plots above. (Wilcoxon, P=0.747, R2 = 0.021). **(C, D)** Comparison of α diversity (as accessed by Chao1 index and Simpson index) based on the genus profile in two groups for Chao1 index, P=0.73; for Simpson, P=0.86. P values are from Wilcoxon test. **(E)** Boxplot of 22 differentially enriched species across Infected and Uninfected group based on Lefse.

First of all, rarefaction curve analysis was carried out to evaluate whether the sequence size or sample size was complete or incomplete. The curve in each group is near smooth when the sequencing data are great enough with few new species undetected (**Figure 3A**). To evaluate the diversity change and exclude the confounding factors like gender, batch effect, underlying disease, we performed PerMANOVA analyze and the variance explained by gender (P=0.02, **Supplementary Table S6**) were greater than by other potential cofounders and disease status (P=0.22 and 0.13, respectively, **Supplementary Table S6**). Next, we conducted an analysis of microbial changes at the community level between infected group and uninfected group among 42 male samples (30 infected and 12 uninfected). The Chao1 index and Simpson index analyzes showed that there was no significance in infected group versus uninfected group at the genus level (PerMANOVA, P=0.073 and 0.86, respectively, **Figures 3C, D**). The Chao1 median index was higher in infected group which may indicate bacteria are more likely to increase in infected group. Beta diversity based on weighted principal coordinate analysis (PCoA) revealed that there was no different separation in infected group versus uninfected group (PerMANOVA, P=0.75, **Figure 3B**). Then, we performed a Lefse analysis to identify differentially enriched taxa in both groups. By LDA>3 cutoff, we got 65 significant biomarkers (**Supplementary Table S7**) in total and 22 were at species level presented in **Figure 3E**. Three were observed in the infected group (*E. clbacae, K. oxytoca* and *C. freundii*) and nineteen in uninfected group as follows: *C. acnes, Human polyomavirus 2, Ralstonia insidiosa, Staphylococcus hominis, Staphylococcus epidermidis, Ralstonia pickettii, Burkholderia multivorans, Staphylococcus warneri, Moraxella osloensis, Burkholderia cepacia, Gordonia bronchialis, Gordonia paraffiniivorans, Corynebacterium tuberculostearicum, Corynebacterium pseudogenitalium, Malassezia restricta, Methyotenera mobilis, Paraburkholderia fungorum* and *Burkholderia pyrrocinia*. Most of the uninfected enriched microorganisms were from environment.

**Figure 4:**
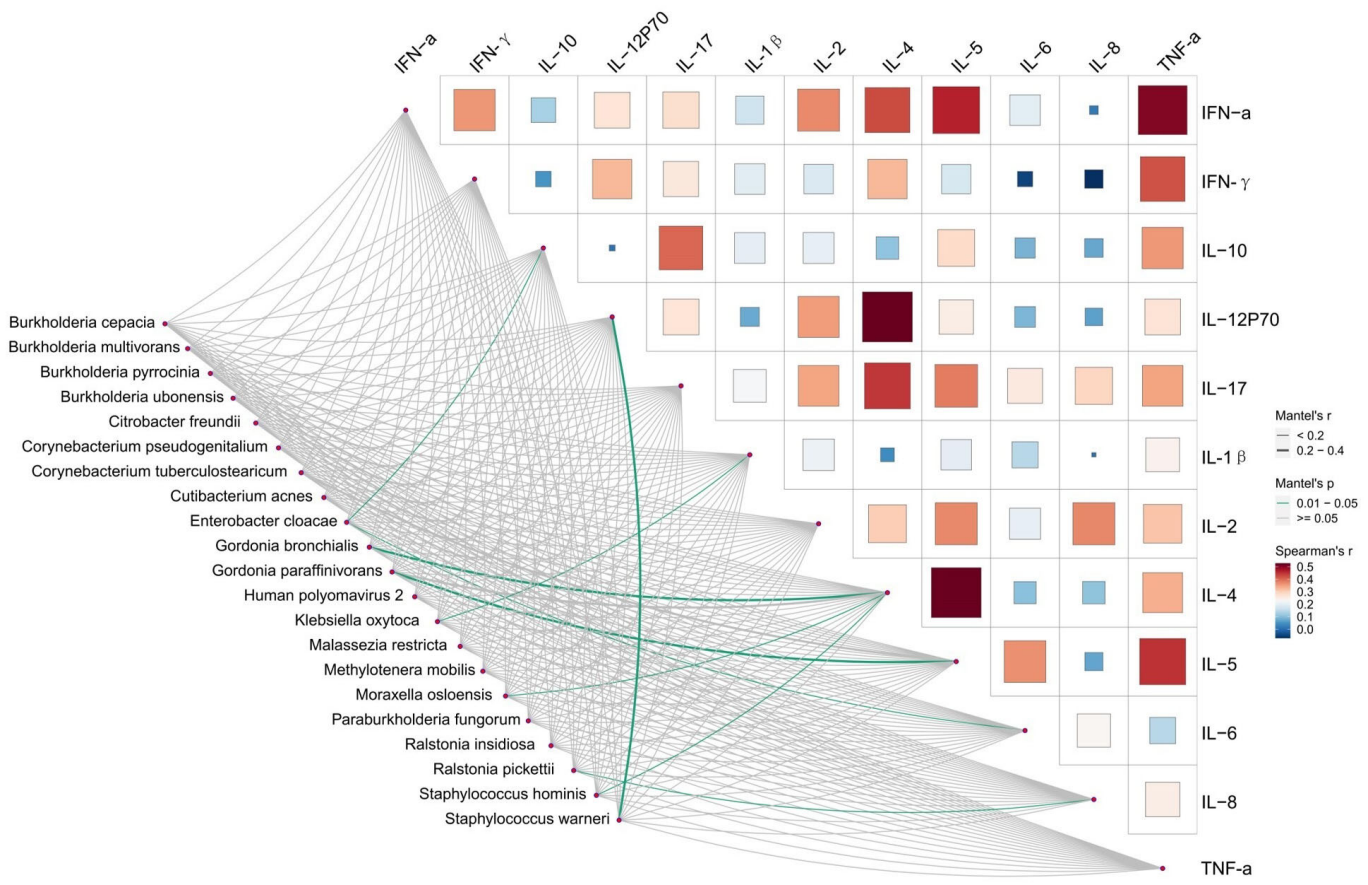
Relationships between Inflammatory factors and microbiome diversity. Pairwise comparisons of Inflammatory factors with a color gradient denoting Spearman’s correlation coefficients. Taxonomic groups were related to each Inflammatory factors by mantel test. Green line indicates the significant relationship and gray line indicates the unsignificant relationship. Line width means the weight of regression coefficient.

We conducted a correlated analysis between the differentially taxa and clinical variables using Mantel test. As is shown in **Figure 4** and **Supplementary Table S8**, Infected-enriched *E. cloacae* were significantly correlated with IL-6 and IL-10 (IL-10 = 0.16, P< 0.05, IL-6 = 0.18, P< 0.05). Besides, Infected-enriched *K. oxytoca* were significantly correlated with IL-1β (IL-1β=0.15, P<0.05). For Uninfected-enriched biomarkers, the Mantel test result are as follows: *G. bronchialis*: rIL-4 = 0.28, P<0.05; *G. paraffinivorans:* rIL-5 = 0.24, P< 0.05; *M. osloensis:* rIL-4 = 0.13, P<0.05; *R. pickettii:* rIL-8 = 0.09, P<0.05; *S. hominis:* IL-4 = 0.15, P<0.05; *S. warneri:* rIL-12P70 = 0.26, P<0.05). These findings suggested that the Lefse biomarkers were related to ILs level which may be associated with CU infection.

### Virulence factors comparation

**Figure 5:**
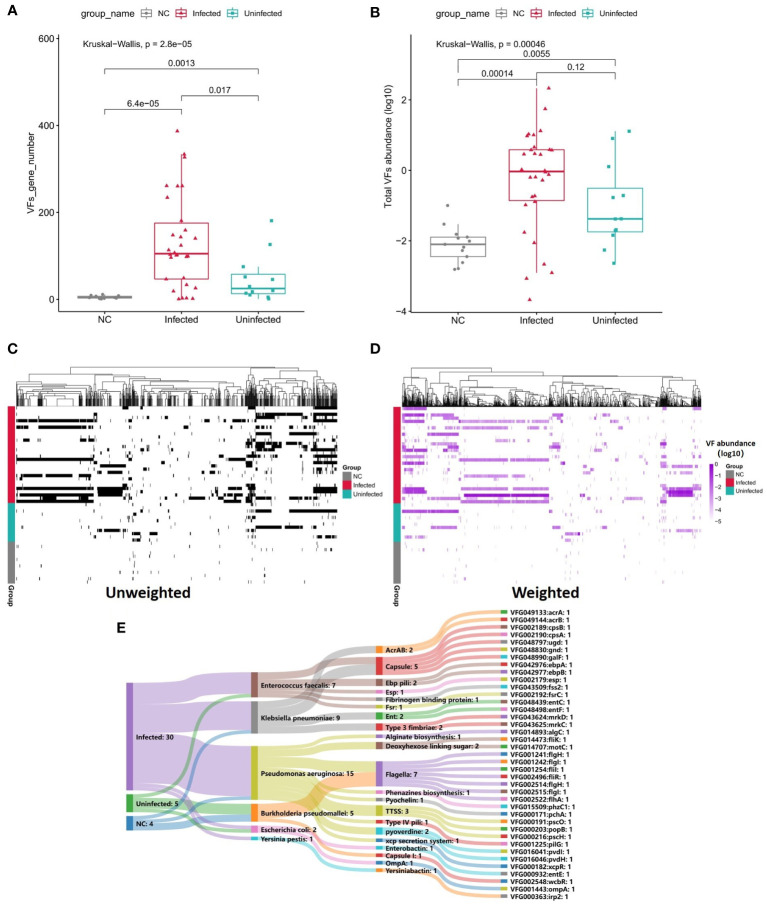
**(A)** Boxplot shows the number of VFs among three groups. P values are from Wilcoxon test. **(B)** Boxplot shows the LOG10-based abundance of VFs among three groups. P values are from Wilcoxon test. **(C)** Heatmap (unweighted) shows the association between VFs and samples. Black shows the identification of the VF while white shows no identification of the VF. **(D)** Heatmap (weighted, MaxAbs method) shows the association between VFs and samples. Shades of purple shows the abundance of the VF, the deeper the color, the higher the abundance. The normalization was through MaxAbs method. That is, the abundance of each gene is divided by the absolute value of the maximum VF abundance in all samples, and then log10 is further taken. VF even abundance=log (VF abundance/|Max VF abundance|). As for VF abundance=0, VF even abundance=-5. **(E)** Sankey map for differential VFs, Based on VF abundance profile, set LDA=3 to screen the enriched VFs in Infected group, Uninfected group and controls, and set VFs annotation information (VFs type, species attribution) in VFDB database.

To survive in a hostile host environment, pathogenic bacteria tend to establish infections by deploying VFs (Ünal and Steinert, 2014). We compared the VFs among three groups. Through unweighted analysis, the VFs gene number were significantly increased in Infected group compared with Uninfected group (P=0.017, **Figures 5A, C**). Through weighted analysis, the abundance of VFs was increased in the Infected group compared with Uninfected group (P=0.12, **Figure 5B**). The abundance of VFs in both Infected group and Uninfected group were significantly more than controls (P<0.05, **Figures 5B, D**). We identified 39 significantly enriched VFs among three groups *via* LEFSE, including 30 VFs in Infected group, 5 VFs in Uninfected group and 4 VFs in controls (**Figure 5E**). In Infected group, most of the enriched VFs were assigned as *Capsule*, *Flagella*, *pyoverdine*, *TTSS* and so on. The main contributors to the enriched VFs were *P. aeruginosa, K. pneumoniae* and *E. faecalis*. In Uninfected group, the enriched VFs were assigned as *Flagella*, *Capsule I*, *Esp* and *Enterobactinthe*. The main contribution to the enriched VFs was *Burkholderia pseudomallei*. In controls, the enriched VFs were assigned as *Flagella*, *AcrAB* and *xcp secretion system*. The main contribution to the enriched VFs was *B. pseudomallei* (**Supplementary Table S9**). In a word, the more VFs the more serious infection.

### ARGs comparation

**Figure 6:**
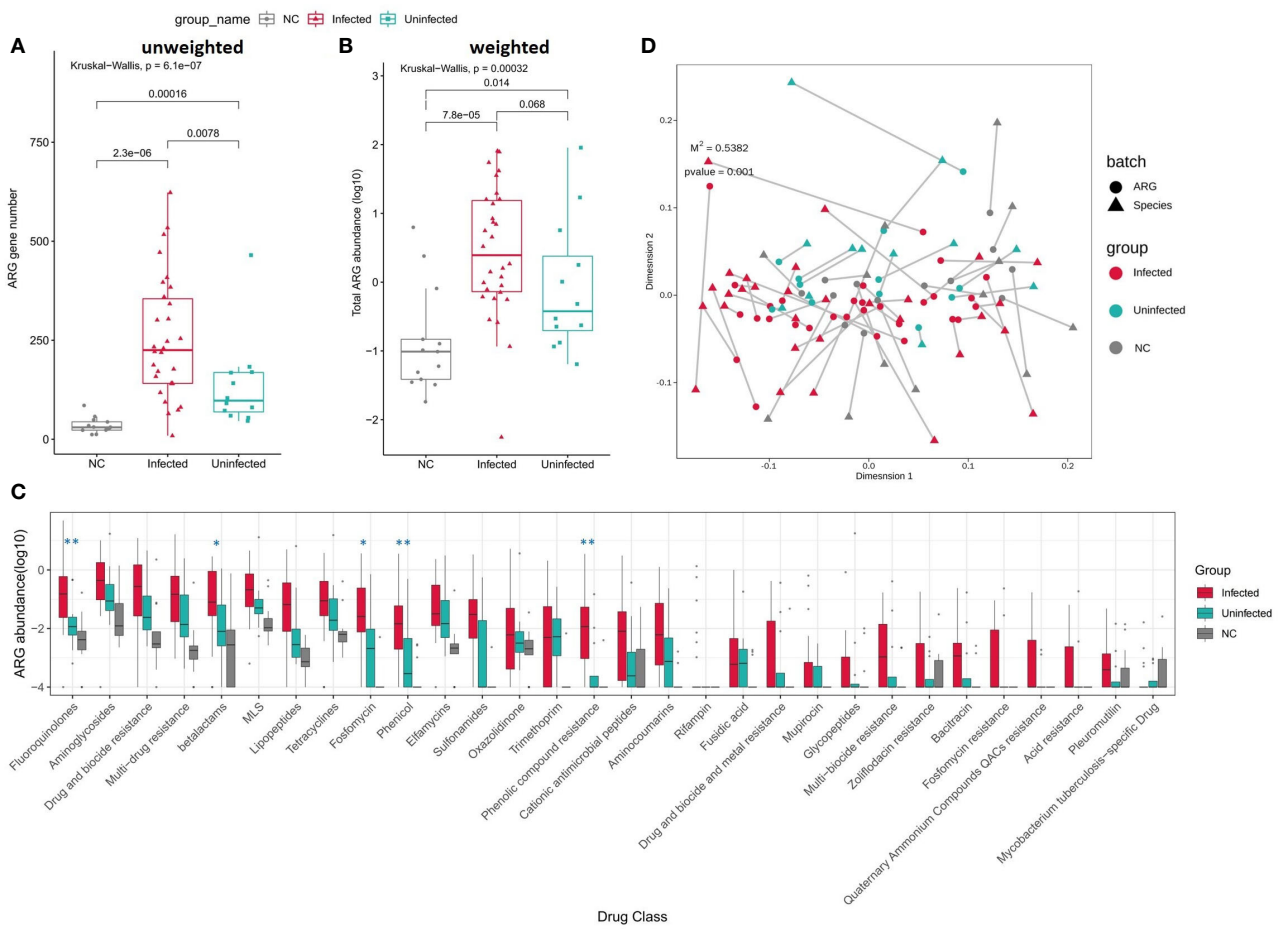
**(A)** Boxplot shows the number of ARGs among three groups. P values are from Wilcoxon test. **(B)** Boxplot shows the LOG10-based abundance of ARGs among three groups. P values are from Wilcoxon test. **(C)** Boxplot shows the LOG10-based abundance of ARGs of each drug class among three groups. (Wilcoxon,*: 0.01<p<0.05,** :p<0.01) **(D)** Procrustes analysis of the correlation between ARG profiles and species community structure on the base of Bray-Curtis matrices.

When comparing to Uninfected group and controls, the numbers of ARGs were significantly increased in Infected group (**Figures 6A, B**). The total abundance of ARGs was also insignificantly increased in Infected group when compared to Uninfected group. The abundance of ARGs assigned to each drug class was also higher in Infected group than Uninfected group. Among them, the abundance of Fluoroquinolones, betalatmas, Fosfomycin, Phenicol, Phenolic compound resistance were significant higher in Infected group (**Figure 6C**). We performed a Procrustes analysis in order to demonstrate the associations between ARGs and microbial composition. The analysis revealed a significant correlation (P = 0.001, 9999 permutations) between ARGs profiles and species-based microbial community composition, with the Procrustes sum of squares M2 = 0.5382 (**Figure 6D**).

## Discussion

In this study, we aim to evaluate the mNSG performance comparing with traditional culture for infected or uninfected patients undergoing CU. Benchmarking clinical culture, the total sensitivity is 91.4% and specificity is 76.3% for mNGS. According to the intersection microorganism profile among urine, swabs and ureteral stent samples, pathogenic organisms in the urine may originate from their surrounding environment. Inflammatory factors like IL-6, IL-1β, and IFN-a or microorganisms biomarkers *C. freundii, K. oxytoca*, and *E. cloacae* need further research for indicating infection and constructing UTI diagnostic model of patients with CU. VFs and ARGs increase in infected group may have bad effect on clinical treatment for patients.

### The incidence and risk factors of UTI

Some underlying host factors are known to exert influence on the etiology of UTI and may complicate UIT, including age, diabetes, spinal cord injury, and catheterization (Ronald, 2003). In our study patients undergoing CU with chronic underlying diseases have a higher risk of infection. There are few studies in existing literatures relating to the rate, risk factors and common pathogens of UTI in patients undergoing CU. Given the large differences in the incidence of UTI and the high rate of asymptomatic bacteriuria reported in the literature (Mano et al., 2014; Ghoreifi et al., 2020; Gayarre Abril et al., 2021), it follows that UTI needs to be clearly defined in order to better define the specific form of UTI in this unique patient population so that we can treat this condition appropriately. In terms of risk factors, it is not yet confirmed if coexisting diabetes increases the risk of UTI. A previous study of 1248 patients with different urinary diversion types showed that the presence of diabetes increased the risk of UTI after RC (Parker et al., 2016). However, another study, involving patients with an orthotopic neobladder (ONB), showed that age, gender, Charlson Comorbidity Index (CCI), perioperative chemotherapy, and diabetes are not associated with UTI (Mano et al., 2014). However, another study showed that none of these were risk factors except for CCI (Clifford et al., 2018). Generally, the incidence of UTI is higher in women. As bladder cancer is known to be more prevalent in males and our study sample size was limited, our results showed that males had a higher risk of infection.

### The relationship between cytokines and UTI

As a family of cytokines, interleukins play a hugely significant part in the regulation of the immune system. Secreted by white blood cells and the epithelial cells of the urinary tract, IL-6 is a highly sensitive and specific biomarker for UTI and is a known response to inflammation (Yang et al., 2021). In the immune response to UTI, IL-6 and IL-8 are activated and released (Klarström Engström et al., 2019); both of these are associated with the severity of UTI (Sundén et al., 2017). Both Sheu (Sheu et al., 2006) and Gurgoze (Gürgöze et al., 2005) confirmed that the serum levels of IL-6 in patients with pyelonephritis were obviously higher than lower urinary tract infection (L-UTI) patients or healthy individuals. In another study. Olszyna reported that the concentrations of IL-8 in patients with positive urine cultures were significantly higher than those of healthy subjects (Olszyna et al., 2001). Another researcher confirmed that serum levels of IL-6 were more sensitive and specific than IL-8 for the diagnosis of pyelonephritis (Sheu et al., 2007). Conversely, Krzemien did not observe a discrepancy between urinary IL-6 and IL-8 concentrations between patients with pyelonephritis and L-UTI (Krzemień et al., 2004). Il-1 β, a sub-type of IL-1, which is found at high levels in various body fluids, such as the serum, urine, and synovial fluid, is known to be involved in the regulation of the immune system. IL-1β is considered as a candidate biomarker to distinguish upper urinary tract infection (U-UTI) from L-UTI (Masajtis-Zagajewska and Nowicki, 2017). Other research has shown that several cytokines (TNF-α, IL-8, IL-6, and IL1B) can significantly promote the growth of uropathogenic *E. coli* (UPEC) (Engelsöy et al., 2019; Demirel et al., 2020). The inhibition of NF-KB can lead to the long-term colonization of UPEC in the bladder; moreover, RELA and NFKB1 can be activated by UPEC colonization (Liu et al., 2015). Similar to these published findings, we found that the levels of IL-6, IL-1β, and IFN-α were significantly increased in the infected group. Further analysis should attempt to correlate these factors with other indicators or changes in the flora in order to better apply these promising biomarkers.

### Statistical analysis of pathogenic detection by clinical culture

Bacteriological cultures of urine are usually positive for aerobic or fast-growing organisms, such as *E. coli* and *E. faecalis* (Foxman, 2014). UPEC utilize multiple chaperone–usher pathway pili tipped with adhesins with diverse receptor specificities to colonize various host tissues and habitats (Kalas et al., 2018). A retrospective analysis of 1133 patients after RC (Clifford et al., 2018) identified the same top three pathogens as we did in the present study; these pathogens also existed in the uninfected group. We therefore speculate that these organisms would multiply and cause infection when the body’s immunity was low or when the balance of the flora was disrupted. In addition, we found that most of the bacteria cultured in urine also existed in skin swabs and ureteral stents, thus indicating that the pathogenic organisms in urine may originate from their surrounding environment. Therefore, on one hand, we need to focus on the skin around the stoma, as this can be colonized by a diverse milieu of organisms depending on the topographical location, endogenous host factors, and exogenous environmental factors relating to the ecology of the skin surface. Usually most of these organisms are harmless or even beneficial to their host unless common skin disorders or some external intervention occurs (Grice and Segre, 2011). If a patient presents with peristomal skin complications, the balance in the flora may be disrupted, thereby increasing the risk of UTI. On the other hand, patient health education should be enhanced; they should be instructed to pay more attention to hand hygiene when changing stoma bags daily. Furthermore, stoma bags should not be used for long periods of time. By performing these simple tasks, it is possible to reduce the risk of bacterial invasion and colonization.

### Potential of mNGS for assisting UTI diagnose

Many reports have shown that mNGS has higher levels of sensitivity than traditional culture for certain sample types, such as blood, cerebrospinal fluid, and bronchoalveolar lavage fluid (Blauwkamp et al., 2019; Liu et al., 2019). However, few studies have attempted to use mNGS on urine samples and compared results to traditional culture methods. In our study, the total sensitivity is 91.4% for mNGS while the specificity is just 76.3%.For example, *E. faecalis*, possibly colonized bacteria in urine, Top1 false positive species in our study (Table S5) which is positive just in 5 samples for culture, but in mNGS besides the 5sample, another 28 samples are positive and Infected: Uninfected is 20:8 which is possibly be explained by culture false negative. The other mNGS false positive microorganisms, like *Shigella sonnei*, just have the same phenomenon. Totally,78% false positive cases happen in infected group, and is accepted by clinician which suggests that mNGS could have a high potential for assisting UTI diagnose. What’s more, mNGS can comprehensively provide a wide range of data on the status of the microenvironment in the urinary tract along with data relating to genotype drug resistance in the diagnosis of UTI (Dixon et al., 2020).

### A comparison of virulence factors

The reason why pathogens can invade a host and reproduce is due to the complex coordination of VFs. Bacterial virulence factors are usually proteins that enable pathogens to parasitize a host, including gene products associated with adhesion, invasion, secretory systems, toxins, and iron uptake systems (Chen et al., 2012). These virulence factors play a role in bacterial adhesion, invasion, and escape from the host’s immune defense, thus enabling them to survive, replicate, and even form and maintain biofilms in the host (Ruer et al., 2015; Mühlen and Dersch, 2016). In the infected group, we found the significant increase of VFs abundance and most of them belonging to *P. aeruginosa*, *E. faecalis*, *K. pneumoniae* including TTSS, HSI.I, Phenazines, and Pyochelin, and VFs in the infected group were mainly related to the secretion system and iron absorption. The secretory system is one of the secretory pathways for VFs and plays a significant role in bacterial pathogenicity. The specific proteins produced by these VFs can be directly introduced into host cells to cause infection. Iron is an indispensable element for the growth of most organisms. Studies have indicated that an increase in the efficiency of the bacterial iron intake system to obtain iron is the main factor responsible for an increase in bacterial virulence; the pathogenicity of bacteria is strongly related to the ability of bacteria to take up iron (Shon et al., 2013). The analysis revealed the enriched VFs in the infected group were mostly *P. aeruginosa* and *E. faecalis* in our study. A previous study demonstrated that the main mechanism responsible for the action of *P. aeruginosa* was intimately associated with the multitype secretion system it encodes, including the type II secretion system (T2SS), type III secretion system (T3SS), type VI secretion system (T6SS), and quorum sensing (QS) system (Golovkine et al., 2017). The 6.3 Mbp genome of *P. aeruginosa* also provides a richer gene pool, and greater virulence, than other pathogens; this is the reason why the virulence genes in the infected group belonged to *P. aeruginosa*. Generally, *E. faecalis* is related to polymicrobial infections of the urinary tract and surgical wound sites. Moreover, the secretion of L-ornithine by *E. faecalis* can stimulate *E. coli* biofilms to grow and survive. Furthermore, under iron limiting conditions, L-ornithine be used by *E. coli* to synthesize the enterobacterium siderophore, thus resulting in the growth of *E. faecalis* and the formation of biofilm (Keogh et al., 2016).

### Drug resistance

Technologies such as NGS are expanding our abilities to detect and study antimicrobial resistance (Boolchandani et al., 2019). Whole-genome sequencing (WGS) studies of *E. coli*, *Enterococcus* spp., *K. pneumoniae*, and *Salmonella enterica serovar Typhimurium* have proved that it is possible to predict resistance phenotypes either by rule-based strategies or by machine learning (Kos et al., 2015; Khaledi et al., 2020). mNGS can improve the detection of pathogens that can easily be missed by clinicians or by traditional culture tests (Ramachandran and Wilson, 2020). Resistance phenotype could be mediated by protein homolog model features, like betalactams *KPC* conferring resistant to carbapenem, protein variant model features, like *P. aeruginosa oprD* variants conferring resistant to carbapenem and *gyrA* variants conferring resistance to quinolones, protein overexpression model features, like efflux pump complex *MexZ* conferring antibiotic resistance to erythromycin in *E. coli*. After infection, ARGs belonging to several drug-class are increased in infected group including those clinical concerned carbapenemase/ESBL genes among Gram-negative bacteria or mecA/C among *S. aureus* and in the future we could possibly use mNGS to monitor those in ARGs profile of CU patients so as to rationally guide the use of antibiotic.

## Conclusion

mNGS, with high sensitivity, could provide a more accurate and fast evidence for helping UTI diagnosis in patients with CU and allow us to monitor the microbial, VFs and ARGs changes in the urine of these patients and improve treatment.

### Limitation

We acknowledge the limitations of our research. First of all, the population of patients show a less diversity and representativeness owing to the subjects were obtained from one hospital. Secondly, there is a lack of unified definition of UTI, especially for complex situations or special populations, such as Cu patients. UTI needs to be clearly defined to better describe the actual UTI rate and treat it in time. The study population have a recurrent UTI with a high incidence, but we didn’t perform a longitudinal study, which is one of the directions that can be studied in the future.

## Data availability statement

The datasets presented in this study can be found in NCBI Sequence Read Archive (SRA), and the accession number is PRJNA779226.

## Ethics statement

The studies involving human participants were reviewed and approved by Ethics Committee of the First Affiliated Hospital of Nanchang University. The patients/participants provided their written informed consent to participate in this study.

## Author contributions

RH wrote the manuscript, and RH and QY both performed and completed the whole experiment, and they were also responsible for data collection. JG analyzed the data and participated in the discussion of drug resistance genes. YL, XJ, LT, and YC contributed to conception and design of the study. YL, LT, and YC directed and supervised all of the research. All authors contributed to the article and approved the submitted version.

## Supplementary material

Supplementary Fig 1
**A**, **B**, Composition ratio of the infected and uninfected samples considering underlying diseases influence (A) and gender (B).

Supplementary Fig 2 Boxplot of 6 significantly different Urinalysis and cytokines in serum (Wilcoxon, *: 0.01<p<0.05, ** :0.001<p<0.01, ***:0.0001<p<0.0001).
