# Supplementary figures and images for: Application of metagenomic next-generation sequencing in the diagnosis of urinary tract infection in patients undergoing cutaneous ureterostomy
Source: Front Cell Infect Microbiol. 2023 Jan 27;13:991011. doi: 10.3389/fcimb.2023.991011 (PMC9911821; doi:10.3389/fcimb.2023.991011)

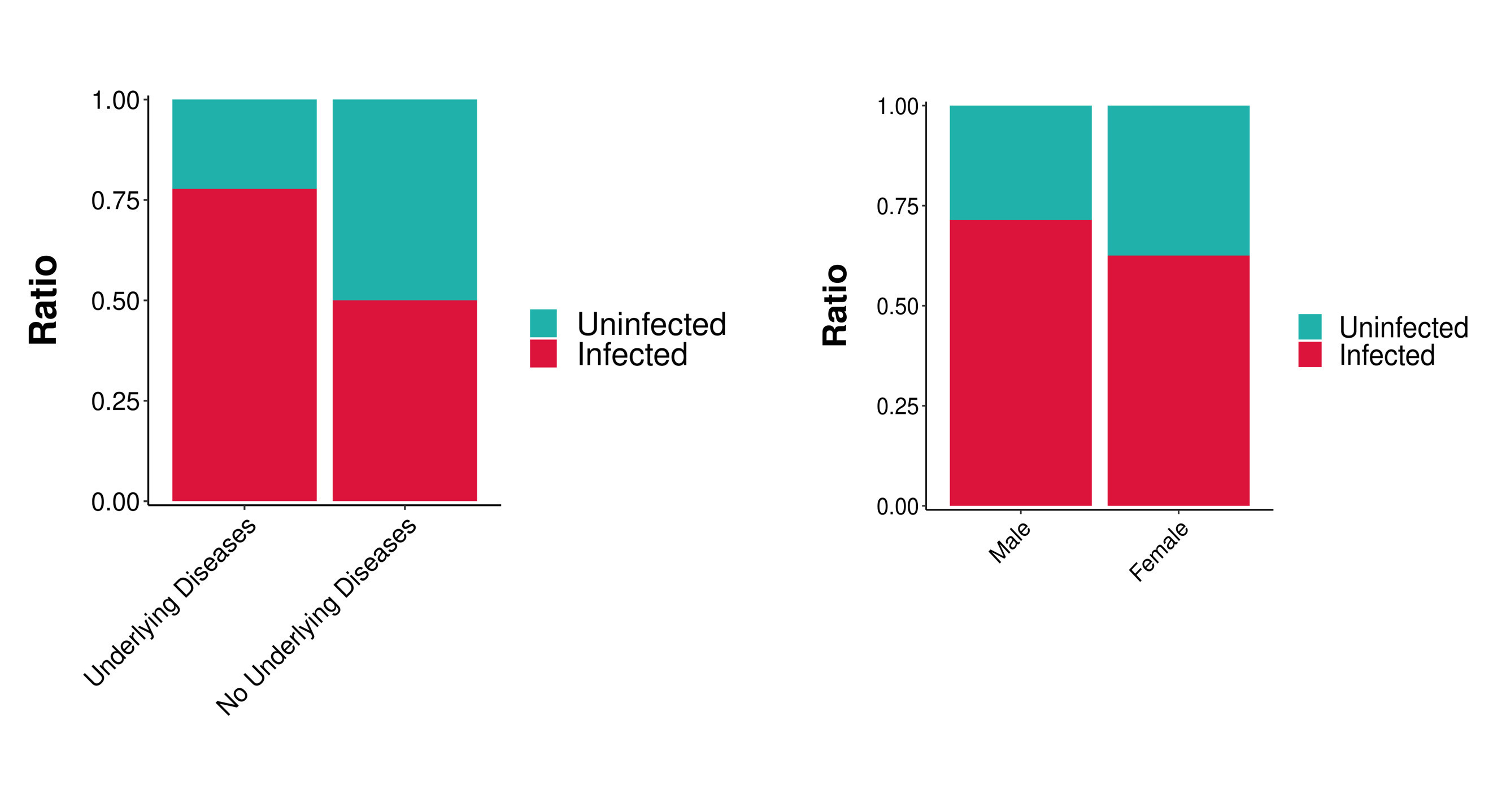

Supplement: Supplementary Fig 1 — A, B, Composition ratio of the infected and uninfected samples considering underlying diseases influence (A) and gender (B). [file Image_1.tiff]

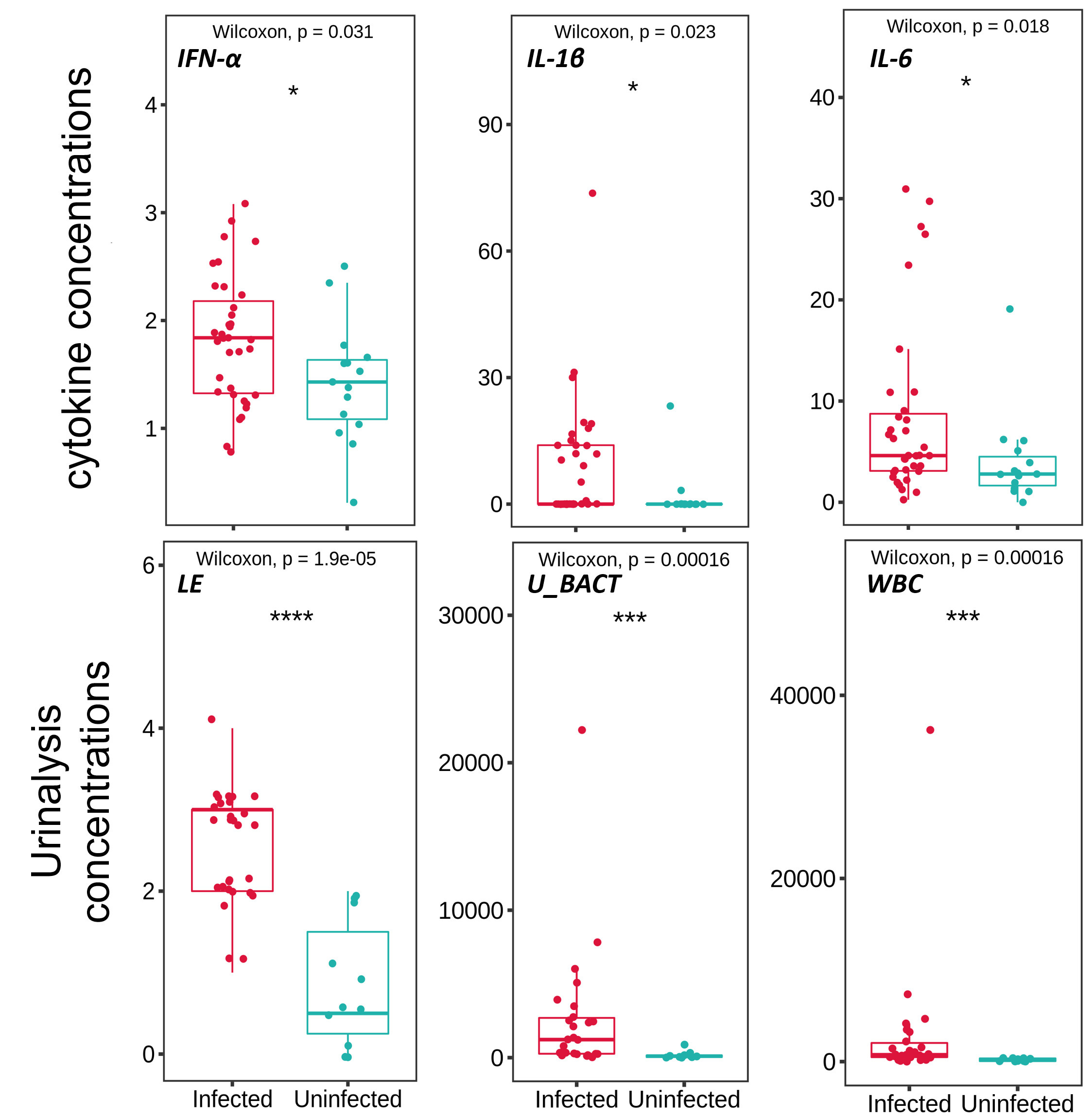

Supplement: Supplementary Fig 2 — Boxplot of 6 significantly different Urinalysis and cytokines in serum (Wilcoxon, *: 0.01<p<0.05, ** :0.001<p<0.01, ***:0.0001<p<0.0001). [file Image_2.tiff]
